# Investigating Childhood Leukemia in Churchill County, Nevada

**DOI:** 10.1289/ehp.9022

**Published:** 2006-11-30

**Authors:** Carol S. Rubin, Adrianne K. Holmes, Martin G. Belson, Robert L. Jones, W. Dana Flanders, Stephanie M. Kieszak, John Osterloh, George E. Luber, Benjamin C. Blount, Dana B. Barr, Karen K. Steinberg, Glen A. Satten, Michael A. McGeehin, Randall L. Todd

**Affiliations:** 1 National Center for Environmental Health, Centers for Disease Control and Prevention, Atlanta, Georgia, USA; 2 Nevada State Health Division, Carson City, Nevada, USA

**Keywords:** ALL, AML, Churchill County, cancer cluster, environment, Fallon, leukemia, tungsten

## Abstract

**Background:**

Sixteen children diagnosed with acute leukemia between 1997 and 2002 lived in Churchill County, Nevada, at the time of or before their illness. Considering the county population and statewide cancer rate, fewer than two cases would be expected.

**Objectives:**

In March 2001, the Centers for Disease Control and Prevention led federal, state, and local agencies in a cross-sectional, case-comparison study to determine if ongoing environmental exposures posed a health risk to residents and to compare levels of contaminants in environmental and biologic samples collected from participating families.

**Methods:**

Surveys with more than 500 variables were administered to 205 people in 69 families. Blood, urine, and cheek cell samples were collected and analyzed for 139 chemicals, eight viral markers, and several genetic polymorphisms. Air, water, soil, and dust samples were collected from almost 80 homes to measure more than 200 chemicals.

**Results:**

The scope of this cancer cluster investigation exceeded any previous study of pediatric leukemia. Nonetheless, no exposure consistent with leukemia risk was identified. Overall, tungsten and arsenic levels in urine and water samples were significantly higher than national comparison values; however, levels were similar among case and comparison groups.

**Conclusions:**

Although the cases in this cancer cluster may in fact have a common etiology, their small number and the length of time between diagnosis and our exposure assessment lessen the ability to find an association between leukemia and environmental exposures. Given the limitations of individual cancer cluster investigations, it may prove more efficient to pool laboratory and questionnaire data from similar leukemia clusters.

## Introduction

Acute lymphocytic leukemia (ALL) is the most commonly diagnosed pediatric cancer in the United States (American Cancer Society 2005; Linet et al. 1999; Sandler and Ross 1997). Established risk factors such as ionizing radiation and prenatal exposure to volatile organic compounds (VOCs) do not explain most ALL diagnoses (Belson et al. 2006; Greaves 1997; Linet and Cartwright 1996). For the most part, clustering of children diagnosed with ALL has been attributed to chance (Boyle et al. 1996; Caldwell 1990; Caldwell and Heath 1976). However, when a county of 26,000 people incurred more than a dozen leukemia diagnoses during a 4-year period in which fewer than two cases were expected, an investigation seemed warranted [Nevada State Health Division (NSHD) 2001; Steinmaus et al. 2004).

### Potential environmental exposures

The city of Fallon (population 8,000) is the only urban center in Churchill County, Nevada. The Naval Air Station–Fallon (NAS Fallon) contributes an additional 3,000 residents to the city’s population. The local community identified four major areas of concern related to environmental exposures. A pipeline runs through downtown Fallon, delivering a continuous supply of JP-8 jet fuel to the base. Both municipal and NAS water supplies have historically reported naturally occurring arsenic levels that exceed U.S. Environmental Protection Agency (U.S. EPA) standards (Welch and Lico 1998; Welch et al. 1989). Fallon is also a center for melon and alfalfa production, and agricultural pesticides are applied to fields surrounding the city. Two facilities related to tungsten refining and use are located in Churchill County: An administrative center and laboratory are in downtown Fallon, and a tungsten carbide processing plant is 11 miles north of Fallon [Agency for Toxic Substances and Disease Registry (ATSDR) 2003].

### Initial identification of the cluster

In July 2000, an astute local health care provider notified state health officials that several Churchill County children had recently been diagnosed with leukemia. In general, state cancer registries experience significant reporting lag, so state health officials conducted active case finding to verify leukemia occurrence. By February 2001, the NSHD had identified 12 children who had been diagnosed with leukemia since 1997 and who had lived in Churchill County before their diagnosis. The state convened an expert panel composed of national cancer specialists, epidemiologists, and public health officials to review these cases. The panel recommended that the state epidemiologist request technical assistance from the Centers for Disease Control and Prevention (CDC) to investigate whether ongoing exposure to environmental contaminants in Churchill County might be endangering human health (Robison et al. 2001). Two new cases were added to the state’s list shortly after the expert panel report was completed, bringing the number of cases included in the state’s investigation to 14.

### Multiagency response

The NSHD responded to recommendations of the expert panel by approaching the CDC for assistance in Churchill County. Beginning in March 2001 the CDC led a multiagency effort to conduct a comprehensive cross-sectional exposure assessment. In Table 1 we list the state and federal agencies that were involved and identify the responsibilities of each agency. All involved agencies were represented at community meetings held throughout the study period to provide updates on the status of the study. During the course of the investigation, all biologic and environmental laboratory results were reviewed as they were received and were collectively assessed for risk by multidisciplinary expert panels (CDC 2004); this joint review was conducted to fulfill the CDC’s promise to the community that any results suggesting the possibility of an ongoing health risk, regardless of known significance to the cluster analysis, would be released to the community immediately rather than waiting until the end of the study.

## Materials and Methods

### Case definition and selection of comparison families

**Figure 1:**
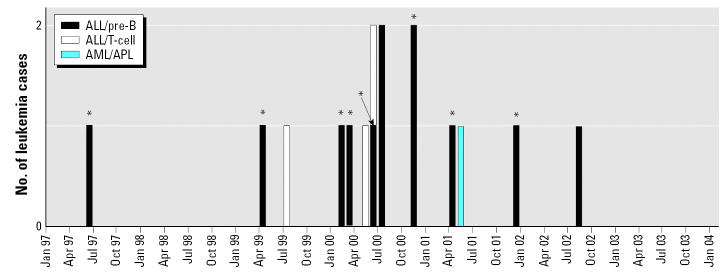
Temporal distribution of the 16 children who had lived in Churchill County, Nevada, and were diagnosed with leukemia between June 1997 and August 2002. Cancer type and cell type are defined. One case declined participation in the study, and one was diagnosed outside the study period. The nine individuals fitting the restricted case definition are indicated by asterisks (*).

We defined a case family as the child diagnosed with leukemia and all other people currently living in the child’s home (i.e., all siblings, parents, guardians, and other adults), as well as biologic parents who were not living full time in the case child’s home and for whom contact information was available. At the time the CDC study was initiated, 14 case children had already been identified by NSHD. All 14 of the families of these children were approached for inclusion in the CDC study; 13 of these families agreed to participate. One of the case children from these families died before our study began. A 15th child was diagnosed and enrolled in the CDC study in December 2001. A 16th child was diagnosed after 31 December 2001, the end of the study enrollment period. In all, 15 case families were eligible to participate, and 14 were enrolled in the study. In Figure 1 we describe the date of diagnosis and leukemia cell type for all 16 children diagnosed with leukemia. Of the 16 cases, 10 (62.5%) were residents of Churchill County at the time of diagnosis.

Because leukemia etiology (e.g., genetic predisposition, prenatal exposure, postnatal exposure) may be related to age at diagnosis, leukemia type [ALL, acute myelocytic leukemia (AML)], cell type (B-cell, T-cell), or geographic location, we constructed a stricter case definition based on age (0–6 years at time of diagnosis), diagnosis (ALL pre-B), and duration of residence in Churchill County (at least 6 months before diagnosis). Nine children met the restricted case definition (Figure 1).

Comparison families from Churchill County were identified through random-digit dialing. Fifty-five (43.6%) of the 126 comparison families identified as eligible were enrolled. We attempted to match four comparison children to each case child by birth year (in 2-year increments) and sex. The comparison child was required to be cancer free at the date of the case child’s leukemia diagnosis. We defined a comparison family as the matched comparison child and that child’s parents, guardians, or other care-taking adults living full time in the comparison child’s home.

A total of 205 participants representing 14 (of the 15 eligible) case families and 55 comparison families were enrolled and asked to complete mailed questionnaires, participate in personal interviews, donate biologic samples, open their homes to environmental sampling, and provide information about previous local residences that could be sampled. We enrolled 4 comparison families matched to the case family that declined participation; these data were included in the cross-sectional analysis but not in the case-comparison analysis. Some members of one enrolled case family chose not to participate; the information from that case family is included in the case-comparison analysis. The study described here complies with all applicable requirements of the U.S. regulations including institutional review board (IRB) approval. All participants signed consent or assent forms that had been approved as part of the IRB review of the protocol for this study (CDC 2003a).

### Biologic samples

Beginning in August 2001, all participants were invited to visit a CDC clinic in Fallon, where blood, urine, and cheek swab samples were collected in a highly controlled setting (CDC 2003b) and personal interviews were administered. The biologic samples were aliquoted on site in a biological safety cabinet to maintain sample integrity and to keep the samples free from chemical contamination and infectious agents. These samples were sent daily to CDC laboratories in Atlanta, Georgia, where they were analyzed for essential and toxic metals (CDC 2003b), nonpersistent pesticides (Baker et al. 2004; Bravo et al. 2002; Olsson et al. 2004), persistent pesticides and polychlorinated biphenyls (PCBs) (Barr et al. 2003), VOCs (Cardinali et al. 2000), and markers of past exposure to infectious diseases. Concentrations less than the limit of detection (LOD) were assigned a value equal to the LOD divided by the square root of 2.

We considered both creatinine-corrected and non-creatinine-corrected results for analyses of urine samples and lipid-adjusted and non-lipid-adjusted results for analyses of blood samples. In this article we present non-creatinine-corrected results for metal and nonpersistent pesticide levels in urine samples because creatinine correction may not be reliable in children (Barr et al. 2005; O’Rourke et al. 2000) and because non-creatinine-corrected comparison values for most analytes were available in the second National Report on Human Exposure to Environmental Chemicals (second NER) (CDC 2003d). We present lipid-adjusted results for persistent pesticide and PCB levels in serum samples, and results not adjusted for lipids for VOCs analyzed in blood samples.

### Environmental samples

Using standardized protocols (CDC/Nevada Department of Environmental Protection 2005), the Nevada Department of Environmental Protection collected samples of indoor air, play yard soil, and household dust from case families’ current and previous residences in Churchill County, current residences of all comparison families, and previous residences for one randomly selected comparison child for each case child. Tap water samples from all of these residences were collected by the U.S. Geological Survey (USGS). Environmental samples were successfully collected from all 11 homes of the case families currently living in Churchill County, all 8 previous homes in Churchill County in which case families had lived before the date of birth of the case child (i.e., during pregnancy), current homes of all 55 comparison families, and 6 previous residences of 15 comparison families. Of 36 previous residences eligible for sampling, 8 case homes and 14 comparison homes were not accessible (e.g., house was destroyed or vacant, or current owner was nonresponsive). Current and previous residences outside of Churchill County were not sampled. Household samples were tested for heavy metals, persistent and nonpersistent pesticides, PCBs, VOCs, radon, and radionuclides. Environmental samples were analyzed by the USGS, U.S. EPA Region 9, the Nevada Department of Agriculture, and several contract laboratories. In this article we present results of environmental sample analysis only when they are relevant to biologic sample results.

### Statistical analysis

In our primary analysis, we used univariate statistics to describe each of the exposures analyzed. For continuous variables, we used geometric means and selected percentiles to summarize the range and distribution of the data for the various subpopulations of interest. We calculated geometric means [with 95% confidence intervals (CIs)] assuming that the data approximated a log-normal distribution, and only when the proportion of results above the LOD was at least 60%. The estimates of the mean and 95% CI are based on a statistical model that controlled for the possible correlation of observations within a family (i.e., a variance components model), when appropriate. Categorical variables, frequency counts, and percentages are presented as summary statistics for the subpopulations of interest. In the secondary analysis, we compared exposure among the case and comparison populations using conditional logistic regression; we initially analyzed all cases and their corresponding controls, and then only those cases and their corresponding controls meeting the restricted case definition.

For categorical exposure variables, odds ratios (ORs) are used to assess the association between disease and exposure between two specific levels of the categorical variable. For continuous exposure variables, the ORs are based on data that were standardized before analysis. The exposure measures were standardized by dividing each individual response by the standard deviation observed among the entire study population. Because of the limited sample size, ORs were not adjusted for potential confounders. The LogXact software from Cytel Corporation (version 4.0; Cytel Software Corp., Cambridge, MA) was used to fit the conditional logistic regression models.

For this investigation, many of the logistic regression models compared current levels of exposure among the case and comparison populations. Because both populations were sampled after diagnosis of leukemia among the case population, treatment, past diagnostic procedures, changes in behavior, and changes in chemical exposures over time may all have significant, although immeasurable, impact on the relationships that were explored in our secondary analysis.

### Tungsten follow-up

When preliminary study results suggested unusually high levels of tungsten in urine and water samples collected from Churchill County, we decided to expand our study to include tungsten measurements in three other Nevada communities to determine if the findings in Churchill County were unique or represented levels characteristic of the region. The towns of Lovelock, Yerington, and Pahrump were chosen for a cross-sectional tungsten exposure based on hydrogeologic criteria and history of tungsten mining (CDC 2003c). A geographically random sample pool was selected in each town, including 30 households each from Yerington and Pahrump and 11 households from Lovelock. For sample selection, coordinate grids were superimposed over maps of a given city. A random list of X and Y grid coordinates was generated; the first 30 coordinates falling within the city limits were selected, and the household closest to each coordinate was approached for recruitment. If the first house refused participation or was ineligible, the next closest house was approached. The eligibility criteria for households are residence in the city for at least 1 month before the interview and the presence of one adult and one child younger than 18 years who consent to participate in the study. Eligibility criteria included residence for at least 1 month before the interview. Environmental sampling included tap water, floor dust, and yard soil from each household. Urine samples were collected from one adult and one child younger than 18 years in each family. A total of 141 participants were recruited, ranging in age from 2 to 65 years (children younger than 18 averaged 9.4 years of age and adults averaged 38.9 years of age). Sample collection procedures and their analytical parameters were identical to those used to measure tungsten in the Churchill County study.

## Results

Data collected in Churchill County included responses to 500 questionnaire items, levels of 139 chemicals, and eight viral markers measured in blood and urine samples, including genetic analysis of DNA specimens from whole blood collected from 205 people in 69 families. Levels of more than 200 chemicals were measured in air, water, soil, or dust from almost 80 homes. Among our 69 study children (14 case and 55 comparison), 34 were female and 61 were white. There were no significant differences in proportions of sex, race, and ethnicity between case and comparison children.

Our primary analysis was a cross-sectional study that included biologic measurements. Our secondary analysis compared questionnaire information, biologic values, and environmental findings between case and comparison children and families. We recognized that the primary and secondary analyses of hundreds of questionnaire and laboratory data points would, by sheer probability, result in some statistically significant findings due to chance occurrence. Therefore, we reviewed all results in terms of biological plausibility and also rigorously sought the opinions of panels of experts who reviewed the results of the many data outcomes. Enrolled case children (7 girls and 7 boys) ranged in age from 2 to 19 years at diagnosis; case and matched comparison children (27 girls and 28 boys) ranged in age from 3 to 20 years at time of sample collection. In this article we present results relating to *a*) one environmental contaminant found to be unusually elevated and therefore a potential and ongoing health risk (i.e., tungsten); *b*) lifestyle and demographic factors (e.g., birth weight, breast-feeding) associated with leukemia or exposures to known or suspected carcinogens (e.g., VOCs, arsenic, ionizing radiation, pesticides); and *c*) findings that were statistically significant (e.g., parental age) or of community interest (e.g., military status). Complete results are available at http://www.cdc.gov/nceh/clusters/Fallon (CDC 2004), and genetic results will be presented elsewhere (Steinberg et al. 2006).

### Tungsten

The median tungsten level found in urine samples from the entire study population was 0.97 μg/L (geometric mean, 1.19 μg/L), compared with 0.07 μg/L (geometric mean, 0.08 μg/L) in the 1999 National Health and Nutrition Examination Survey (NHANES), the most current population reference available in August 2002 or in the second NER (CDC 2003d). In our secondary analysis, median levels were similar for case and comparison children (1.93 and 2.35 μg/L) and case and comparison families (0.61 and 0.62 μg/L). Almost 80% of the Churchill County participants had tungsten levels above the 95th percentile (0.48 μg/L) reported in the second NER. The level of exposure to tungsten that may cause health effects is not known. The CDC successfully petitioned the National Toxicology Program of the National Institute of Environmental Health Sciences, National Institutes of Health, to prioritize research regarding the health effects of tungsten exposure. The CDC contracted with USGS to collect and measure tungsten in tap water samples from participants’ homes. Results ranged from < LOD to 290 μg/L. There is no regulatory limit for tungsten in drinking water.

In the three additional Nevada communities that were sampled for tungsten, 68% of the participants had geometric mean levels of urinary tungsten at or above the 95th percentile second NER levels (Table 2). Yerington, the town most similar to Churchill County with respect to hydrology, geology, and land use, had levels of tungsten in urine and water samples that were statistically similar to Churchill County’s samples. Few household soil and dust samples from the three comparison communities yielded detectable tungsten levels; however, the method used had a high LOD (50 μg/g).

### Arsenic

In our cross-sectional analysis, arsenic levels in urine ranged from < LOD to 1,180 μg/L, with a geometric mean of 34.6 μg/L and median of 37.4 μg/L. A national reference value for urinary arsenic levels is currently unavailable. However, a study of arsenic exposure in Washington State showed urinary arsenic levels ranging from 19.6 μg/L (associated with high exposure) to 9.4 μg/L (associated with low exposure) (Kalman et al. 1990). Furthermore, the 95th percentile for nonrandom samples of the U.S. population is 22 μg/L (CDC, unpublished data). Noncarcinogenic clinical effects are observed at urinary concentrations > 200 μg/L (Hall 1998); however, clinical effects from concentrations < 200 μg/L may be possible. Although not a statistically significant finding, levels of arsenic were nominally higher (*p* = 0.25) in children (geometric mean, 38.9 μg/L) than in their parents (geometric mean, 32.3 μg/L). In our secondary analysis, arsenic levels did not differ significantly (*p* = 0.29) between case children (geometric mean, 29.8 μg/L) and comparison children (geometric mean, 41.3 μg/L) or between case families (geometric mean, 23.9 μg/L) and comparison families (geometric mean, 36.0 μg/L) (*p* = 0.18). Arsenic was also measured in tap water samples collected from 70 current and previous residences. Overall results ranged from < LOD to 874 μg/L (median, 50.9 μg/L). At the time of this study, the U.S. EPA regulatory limit for arsenic in municipal drinking water systems was 50 μg/L; as of 1 January 2006, the regulatory limit is 10 μg/L.

### Known or suspected risk factors

Biologically plausible environmental risk factors of concern in Churchill County included exposure to benzene and other VOCs from JP-8 fuel and also exposure to persistent and nonpersistent pesticides. We analyzed other lifestyle and demographic risk factors that have been implicated in the development of leukemia, including exposure to ionizing radiation (Doll 1995; Miller 1967; Preston et al. 1994), parental age at child’s birth (Dockerty et al. 2001), birth weight (Hjalgrim et al. 2004; Robison et al. 1987), breast-feeding (Kwan et al. 2004), history of allergies (Schuz et al. 1999; Wen et al. 2000a), and parental military service (CDC 1988; Wen et al. 2000b).

### VOCs

We analyzed blood samples for 12 VOCs, including benzene, which is a minor component of JP-8 fuel and gasoline. Most study participants had blood benzene levels below method LODs (0.06 ng/mL). Median VOC levels in Churchill County were similar to those reported in NHANES III (Churchill et al. 2001) and other peer-reviewed reference levels (Table 3). Levels of 2,5-dimethylfuran among the study population were consistent with levels reported in the literature (Ashley et al. 1996) for smokers and nonsmokers. In the Churchill County population, smokers had a median level of 0.08 μg/L and nonsmokers had a median level below the LOD (= 0.024). In our secondary analysis, 7 VOCs were detectable in a high enough percentage of samples to calculate ORs. Exposure to ethylbenzene suggested increased risk for leukemia among case children using the broader case definition (OR 2.67; 95% CI, 1.04–6.84) as well as the restricted case definition (OR 6.13; 95% CI, 1.29–29.00). When comparing case and control families using the broad definition of a case, we found a slightly positive, although not statistically significant, association between leukemia status and exposure to ethylbenzene (OR 1.14; 95% CI, 0.73–1.78). This OR was somewhat lower and still not statistically significant when we analyzed levels of ethylbenzene among case and control families using the restricted case definition (OR 1.08; 95% CI, 0.60–1.94).

Tetrachloroethylene was the only VOC that showed a slightly significant protective OR (0.35; 95% CI, 0.14–0.86) between case and comparison families (including case and comparison children and their family members but excluding siblings). When comparing case and comparison children, however, we detected no statistical differences in VOC levels using either the broad case definition (OR 0.32; 95% CI, 0.05–2.14) or the restricted case definition (OR 0.39; 95% CI, 0.04–3.91).

### Pesticides

Geometric mean levels of 5 of the 31 nonpersistent pesticides that we measured in urine samples were above the reference geometric mean (Table 4); 5 of the pesticides were well below the national reference values, and the rest were very similar to reference levels. In our secondary analysis, none of the nonpersistent pesticides were associated with leukemia risk.

**Figure 2:**
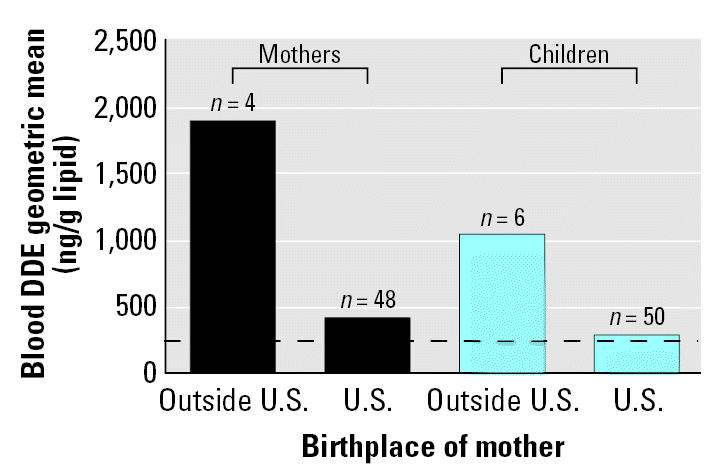
DDE levels measured in blood samples from 52 mothers and 56 children in Churchill County, Nevada, stratified by birth location of mother. Dashed line represents the U.S. mean level of DDE (226 ng/g lipid) (CDC 2003d).

Of the 11 persistent pesticides measured in serum samples, only dichlorodiphenyldichloroethylene [DDE; a breakdown product of dichlorodiphenyltrichloroethane (DDT)] was elevated in our study population (median, 445.3 ng/g lipid; geometric mean, 447.1; range, < LOD to 8169.95) when compared with the second NER (median, 226.0 ng/g lipid; geometric mean, 260). However, when we analyzed DDE levels stratified by birthplace, we found that the elevation persisted only among parents born outside the United States and among children breast-fed by mothers born outside the United States (Figure 2). In the secondary analysis, we found no relationship between leukemia status and exposure to any of the persistent pesticides.

### Lifestyle and demographic factors

#### Ionizing radiation

We used data collected via questionnaire to analyze exposure to ionizing radiation related to medical procedures. We asked whether the study child’s mother received an X ray or other type of radiologic scan, excluding dental X rays, during her pregnancy with the study child. We also asked whether study children were exposed to these same types of radiation before diagnosis for case children and before 30 June 2001 for comparison children. Three mothers (4.7%) reported having an X ray or radiologic scan during pregnancy with the study child; this exposure suggested a nonsignificant association for both broad and restricted case definition analyses (OR 3.46; 95% CI, 0.04–274.70). Forty-one children (60.3%) were reported to have had an X ray or radiologic scan before the date of interest. There was no difference in exposure to ionizing radiation among either the broad (OR 0.65; 95% CI, 0.17–2.41) or restricted (OR 0.19; 95% CI, 0.02–1.17) case definition populations.

#### Parental age

Overall median paternal age at time of birth for our study population was 29 years (range, 16–45 years; mean, 29.2 years). The median age of fathers at the time of birth of case children was 33 years and of comparison children was 28 years. Median maternal age at time of birth for the study population was 26 years (range, 15–37 years; mean, 26.3 years). The median maternal age at time of birth of case children was 28 years and of comparison children was 26 years. Using conditional logistic regression to evaluate differences between case and comparison paternal age, we found an association between leukemia diagnosis and fathers being older at the time of the study child’s birth when age was used as a continuous variable (OR 1.14; 95% CI, 1.01–1.29). This parental age difference persisted for the restricted case definition group (OR 1.19; 95% CI, 1.02–1.39). When we analyzed paternal age as a discrete variable (> 40 vs. < 40 years of age) the association was not statistically significant (OR 3.94; 95% CI, 0.25–62.5). We found no association between case status and maternal age.

#### Birth weight, duration of breast-feeding, and immune status

We looked at questionnaire information regarding birth weight, breast-feeding, and physician diagnosis of allergies because the literature has suggested a possible association between these risk factors and leukemia (Belson et al. 2006; Buffler et al. 2005; Sandler and Ross 1997). Median birth weight for all study children was 3,390 g; median for case children was 3,487 g, and 3,359 g for comparison children. We found a nonsignificant (*p* = 0.27) association (OR 1.73; 95% CI, 0.39–8.14) among children with birth weight > 3,500 g (8 pounds). We found a nonsignificant association (*p* = 0.96) among children who were ever breast-fed (OR 1.35; 95% CI, 0.29–8.50). However, no association was seen when duration of breast-feeding was used as a continuous variable (OR 1.00; 95% CI, 0.99–1.01). Case children were less likely than controls ever to have been diagnosed by a physician with an allergic skin rash (OR 0.067; 95% CI, 0–0.46), although this estimate is considered unstable because no case children answered yes to the question.

#### Military service

Nineteen children (6 case and 13 comparison children) had at least one parent who was serving in the military at some point between 1 year before the child’s birth and the case child’s date of diagnosis. Military service during this time period, however, was not statistically associated with leukemia (OR 3.58; 95% CI, 0.72–20.25). When we restricted our analysis to parents who were in the military during 1 year before the child’s birth (6 case and 10 comparison children), and excluded three comparison children who were adopted, we still found a nonsignificant association between leukemia and military service (OR 5.05; 95% CI, 0.96–29.78). We found no particular patterns in jobs held while in military service. Case parents reported job descriptions including construction, clerical, fighter pilot, hydraulic mechanic, aircraft maintenance, and dentistry.

## Discussion

In this study we examined ongoing environmental exposures by collecting and analyzing biologic and environmental samples at a level of detail not previously attempted or achieved in any other cancer cluster investigation. Nonetheless, no exposure consistent with leukemia risk was identified. We found that tungsten and arsenic were elevated in biologic and environmental samples when compared with samples from other populations, regulatory levels, or health-based recommendations, and we subsequently recommended personal and community actions for reducing exposure. We did not find an association between arsenic or tungsten exposure and leukemia, however. Furthermore, exposure to tungsten in Churchill County does not appear to be unique when compared with certain other similar communities in Nevada. Our studies indicated that people living in communities having water sources and geologic formations similar to those in Churchill County may be expected to have tungsten exposures well above those reported as national reference levels. Moreover, the NSHD has not identified any excess leukemia in the three additional communities studied. The National Toxicology Program is currently evaluating the carcinogenicity of tungsten (National Toxicology Program 2003).

Churchill County residents were aware that their water contained high levels of arsenic, but most participants reported personal historical and ongoing behaviors (e.g., drinking only bottled water, installing point-of-use treatment) intended to decrease their exposure. Most participants were surprised by their elevated biological arsenic levels. The city of Fallon and NAS completed construction on a new water treatment facility in April 2004 that should further reduce arsenic levels in tap water.

Our finding of an elevated OR (5.05; 95% CI, 0.96–29.78) for parental military service, albeit not statistically significant, is suspect given that the six military case parents reported such disparate job descriptions. Further, a recent publication found no general increase in childhood leukemia in other U.S. counties with military bases (Steinmaus et al. 2004). Spurious findings are to be expected in any study, such as this one, that makes thousands of comparisons (Rothman 1990; Wartenberg and Greenberg 1992). Military personnel experience more frequent geographic relocations than most U.S. families, but a single cluster investigation cannot evaluate the impact of population mixing theory on leukemia incidence (Kinlen et al. 1990; Kinlen and Stiller 1993). Our results should be included in research that combines data from several cluster investigations.

### Defining a case

AML and ALL are widely recognized as biologically different diseases and are therefore likely to have different etiologies (Kersey 1997). Moreover, even subgroups of ALL, or widely discrepant age groups within a cell-type subgroup, may manifest different treatment responses and disease outcomes (Greaves 2002). Our study may be limited because we included a variety of leukemia types in some parts of our analysis. Similarly, some parts of our analysis are possibly skewed by inclusion of case children who had only recently moved to Churchill County, had been tangentially exposed to the Churchill County environment, or had moved away from Churchill County long before their diagnosis. To overcome these limitations, we conducted separate statistical analyses on a restricted number of cases that appeared to be most alike. Although these subanalyses did not yield meaningful results, we encourage researchers to combine data from this and future leukemia studies to obtain a larger sample size of cases meeting the strict case definition. Other limitations, particularly of the secondary analyses in which we compared case with comparison subjects, reflect the issue of temporality and our limited ability to control confounding factors.

Cancer clusters can occur by chance. However, the statistical process of fully evaluating the role of chance in a suspected cluster occurrence is daunting and lacks standardization, leaving political and emotional pressures to drive cluster investigations. Ideally, a cancer cluster should be evaluated not just on whether it exceeds previous cancer incidence in a specific defined geographical area during a specific time frame but whether it exceeds expected cancer incidence after accounting for the many ways to define “area,” the large number of areas, as well as the many ways that one could choose a time period for evaluation (Wartenberg 2001).

### The importance of peer review

Cancer cluster investigations are highly charged with emotional and political overtones that inevitably challenge the validity of results (Aldrich and Sinks 2002; Neutra 1990; Rothman 1990; Thun and Sinks 2004; Wartenberg 1995). Affected families and communities are frustrated by a lack of conclusive findings, whereas public health investigators are equally frustrated by their inability to identify a causative agent. In an attempt to mitigate these frustrations, we convened several external peer-review and community-based panels to review and deliberate on our results. This ongoing peer review enhanced communication and increased our confidence in the methods and conclusions of this study.

## Conclusions

This large scale, costly, multiagency response in Churchill County was mounted because this cancer cluster greatly exceeded chance expectation. The families of the 14 case children initially included in the state investigation, plus the two subsequently diagnosed cases, made Churchill County one of the largest pediatric leukemia clusters in U.S. history. Even if one limits the case count to include only children with pre–B-cell ALL who were living in Churchill County at time of diagnosis and for at least 6 months before diagnosis, the number of Churchill County children with this disease is extraordinary.

This cluster occurred at a time when newly advanced laboratory methods enabled quantification of environmental contaminants in biologic and environmental samples at levels not previously possible. Nonetheless, the inability of modern science to identify the role of environmental exposures in leukemia incidence reflects the complexity of defining a relationship between exposure and cancer in a community setting. Biologic samples from Churchill County participants are being stored and will be used by investigators, along with samples from other ALL clusters, to look for emerging environmental carcinogens. Future analyses that combine questionnaire information as well as banked biological data from a series of ALL clusters may yield results that could not be obtained in the Churchill County investigation.

## Figures and Tables

**Table 1:** Agencies that assumed primary roles in the cross-sectional exposure assessment of environmental contaminants in Churchill County, Nevada, 2001–2003, including the responsibilities of each agency.

	NSHD	CDC/ATSDR	NDEP	NDOA	USGS	NAS-Fallon	City of Fallon, Mayor
Study design	✓	✓					
Environmental samples							
Collection			✓	✓	✓	✓	
Analysis				✓	✓		
Biologic samples							
Collection		✓					
Analysis		✓					
Results interpretation	✓	✓	✓	✓	✓	✓	
Community meeting	✓	✓	✓	✓	✓	✓	✓

Abbreviations: NDEP, Nevada Division of Environmental Protection; NDOA, Nevada Department of Agriculture.

**Table 2:** Geometric mean tungsten levels for urine and water samples in multiple Nevada communities.

	Geometric mean tungsten level [μg/L (95% CI)]			
	Urine^a^			
Location	Adults	Children	Total	Tap water
Lovelock	0.38 (0.33–0.45)	0.62 (0.50–0.76)	0.48 (0.34–0.68)	0.11 (0.07–0.19)
Pahrump	0.4 (0.38–0.53)	0.56 (0.48–0.66)	0.51 (0.37–0.69)	0.04 (0.02–0.06)
Yerington	1.04 (0.84–1.30)	1.18 (1.00–1.39)	1.11 (0.97–1.27)	3.32 (1.82–6.04)
Churchill County	0.81 (0.56–1.16)	2.31 (1.66–3.22)	1.19 (0.89–1.59)	4.66 (2.98–7.30)

a The geometric mean urine tungsten level for the NHANES national reference population (reported in the second NER) is 0.08 μg/L (0.07–0.09) (CDC 2003c).

**Table 3:** VOCs (μg/L) in the blood of people living in the United States and people living in Churchill County, Nevada.

	United States		Churchill County	
VOCs	Median levels from NHANES III^a^	95th percentile	Median levels of total study population	Percent > U.S. 95th percentile
1,1,1-Trichloroethane	0.13	0.8	NC	0.0
1,4-Dichlorobenzene	0.33	9.2	0.08	0.0
Benzene	0.061	0.48	0.07	1.0
Carbon tetrachloride	NC^b^	NC	NC	NC
Ethylbenzene	0.060	0.25	0.05	2.0
*m*-/*p*-Xylene	0.19	0.78	0.26	2.0
*o*-Xylene	0.10	0.28	0.06	2.0
Styrene	0.041	0.18	0.05	8.0
Tetrachloroethylene	0.063	0.62	0.04	4.0
Toluene	0.28	1.5	0.2	1.0
Trichloroethene	< LOD	0.021	NC	5.0

NC, not calculated.

a From Churchill et al. (2001). VOCs were not reported in the second NER (CDC 2003d).

b Less than 60% of the study population had detectable levels of this chemical.

**Table 4:** Geometric means and 95th percentile levels in a U.S. reference population of nonpersistent pesticides that were detected in urine samples of > 60% of a study population in Churchill County, Nevada, and the geometric mean levels and percent of the Churchill County study population with levels above the U.S. 95th percentile.

	United States		Churchill County	
Nonpersistent pesticide or metabolite	Geometric mean [μg/L^a^ (95% CI)]	95th percentile (95% CI)	Geometric mean [μg/L (95% CI)]	Percent > U.S. 95th percentile
Chlorpyrifos	1.77 (1.56–2.01)	9.90 (7.60–14.0)	2.46 (1.93–3.14)	16.3
Diethylthiophosphate	NC^b^	2.20 (1.70–2.80)	1.04 (0.81–1.33)	29.5
2,4-Dichlorophenol	1.11 (0.88–1.40)	22.0 (17.0–31.0)	1.15 (0.91–1.46)	0.5
2,4,5-Trichlorophenol	NC	16.0 (4.30–39.0)	4.48 (3.64–5.53)	20.3
2-Naphthol	0.47 (0.33–0.68)	15.0 (9.90–19.3)	0.98 (0.73–1.32)	8.4

NC, not calculated.

a Urine levels are not creatinine adjusted.

b Less than 60% of the study population had detectable levels of this chemical.
